# The current malaria morbidity and mortality in different transmission settings in Western Kenya

**DOI:** 10.1371/journal.pone.0202031

**Published:** 2018-08-09

**Authors:** Anthony Kapesa, Eliningaya J. Kweka, Harrysone Atieli, Yaw A. Afrane, Erasmus Kamugisha, Ming-Chieh Lee, Guofa Zhou, Andrew K. Githeko, Guiyun Yan

**Affiliations:** 1 Climate and Health Laboratory, Centre for Global Health Research, Kenya Medical Research Institute, Kisumu, Kenya; 2 Department of Community Medicine, School of Public Health, Catholic University of Health and Allied Sciences, Mwanza, Tanzania; 3 Department of Medical Parasitology and Entomology, School of Medicine, Catholic University of Health and Allied Sciences, Mwanza, Tanzania; 4 Division of Livestock and Human Health Disease Vector Control, Tropical Pesticides Research Institute, Arusha, Tanzania; 5 Department of Medical Microbiology, College of Health Sciences, University of Ghana, Accra, Ghana; 6 Department of Biochemistry and Molecular Biology, School of Medicine, Catholic University of Health and Allied Sciences, Mwanza, Tanzania; 7 Program in Public Health, University of California, Irvine, CA, United States of America; Universidade Nova de Lisboa Instituto de Higiene e Medicina Tropical, PORTUGAL

## Abstract

**Background:**

Passive surveillance of malaria in health facilities remains vital for implementation of control and elimination programs. It is therefore essential understanding current age profile of clinical malaria morbidity, mortality and presentations in areas with variant infection susceptibility. This study aimed at understanding the current malaria morbidity and mortality in Western Kenya.

**Methods:**

Surveillance of clinical and asymptomatic parasitological positivity rates of all malaria suspected patients and school children were respectively determined from June 2015 to August 2016. From 2014 to 2016, register books in hospitals were referred and the confirmed malaria cases in conjunction with total number of monthly outpatient visits (OPD) counted. All registered malaria admissions were counted together with other causes of admissions. Moreover, outcome of malaria admissions in terms of discharge or death was recorded using inpatient charts within the same time frame. Prospective surveillance of severe malaria collected information on clinical features of the disease. Giemsa stained blood slides confirmed existence of malaria parasitemia. Chi-square and analysis of variance tests were used, respectively, to compute proportions and means; then a comparison was made between different age groups, periods, and study areas.

**Results:**

During the survey of asymptomatic infections among school children, overall blood slide positivity ranged from 6.4% at the epidemic prone site to 38.3% at the hyperendemic site. During the clinical malaria survey, school age children (5–14) presented with overall the highest (45%) blood slide positivity rate among those suspected to have the infection at the epidemic prone study site. The survey of all malaria confirmed and registered cases at OPD found 17% to 27% of all consultations among <5 children and 9.9% to 20.7% of all OPD visits among the ≥5 patients were due to malaria. Moreover, survey of all registered causes of admission in hospitals found 47% of admissions were due to malaria. The disease was a major cause of admission in epidemic prone setting where 63.4% of the <5 children and 62.8% of the ≥5 patients were admitted due to malaria (p>0.05) and 40% of all malaria admissions were school age children. Malaria related death rate was highest among <5 years at the hyperendemic site, that is 60.9 death per 1000 malaria <5 admissions. Conversely, the epidemic prone setting experienced highest malaria related death among ≥15 years (18.6 death per 1000 admissions) than the < 15 years (5.7 death per 1000 admissions of the <15 years) (p< 0.001). Surveillance of severe form of the disease found that hyperpyrexia, hyperparastemia, prostration and convulsions as common presentations of severe disease.

**Conclusion:**

Malaria is still the major cause of hospital consultations in Western Kenya with an alarming number of severe forms of the disease among the school aged children at the epidemic prone setting. Mortalities were higher among <5 children years in high infection transmission setting and among ≥15 years in low and moderate transmission settings. Surveillance of asymptomatic and symptomatic malaria along with evaluation of current interventions in different age groups should be implemented in Kenya.

## Background

In spite of the ongoing interventions, malaria remains a major public health threat to tropical and subtropical countries with more burden to sub-Saharan Africa. These interventions include case management using Artemisinin Combination Therapy (ACT) drugs and vector control through large-scale distribution of Long-Lasting Impregnated Net (LLINs), and Indoor Residual Spraying (IRS). Africa continues to shoulder the heaviest burden of all malaria cases; it registers 90% of all malaria cases and 92% of mortalities [1]. In malaria endemic areas, before the scaling up of interventions, the disease accounted for 25–35% of all outpatient consultations, 20–45% of hospital admissions, and 15–35% of all hospital deaths [2]. However, admissions due to malaria has been decreasing following the scaled up interventions in many areas of sub-Sahara Africa [3,4]. Elsewhere in Africa, resurgence of malaria infection has resulted in causing more burden among older children and adults due to a long spell of sustained malaria control [5]. However, severity of clinical malaria can be affected by a number of factors including parasite genetics and host immune factors [6–8]. While hyperparastemia, hyperpyrexia and convulsions used to be the common presenting symptoms of the severe disease among children in epidemic prone areas before intensive interventions [9,10], severe malaria anaemia, and pulmonary oedema were commonly reported features in hyperendemic areas [10]. It has been established that the introduction of LLINs has led to decrement of anaemia among children in Kenya, and therefore interventional pressure could also affect clinical disease presentations [11,12].

Currently, some areas in western Kenya are experiencing changing dynamics of malaria transmission despite the increasing use of insecticide treated nets and other interventions [13–15]. Climatic warming, vector population species shift, and insecticide resistance had been linked with this incident [14,16]. It is important to ascertain epidemiological changes associated with upsurge and sustained high malaria transmission in the context of admission age profiles, clinical presentations of severe disease and case fatality rates. Updated data on the contribution of malaria on the outpatient hospital services is also important especially in these areas that show changing risks of infection transmission. This information will provide better planning and implementation opportunities for the National malaria strategic plan to meet the 2030 Global targets. This study therefore aimed at describing the current morbidity and the related case fatality rates of malaria of three areas with different infection transmission intensity in Western Kenya.

## Ethical consideration

The study was approved by the Ethical Review board of Kenya Medical Research Institute (SSC protocol No.3005) and got permission from local authorities. An informed consent was sought to human participants before they were involved in the study. The parents signed the assent form on behalf of their school children to allow them participate in the study before their assent.

## Materials and methods

### Study area

This study was conducted in three areas with different malaria transmission intensities. The areas included Marani (epidemic prone), Iguhu (mesoendemic) and Kombewa (hyperendemic) [17]. These areas experience long and short rainy seasons favouring vector breeding. The long rainy season starts from March to June and the shorter rainy season begins from October to November. Table 1 describes topographical, malaria endemicity and characteristics of surveyed hospitals and schools.

**Table 1:** Description of the study areas with different malaria transmission intensities in western Kenya.

Category	Marani	Iguhu	Kombewa	Citation
Location	34^0^48ʹE, 0^0^ 35ʹS,	34^0^45ʹE, 0^0^10ʹN,	34^0^30ʹE, 0^0^07ʹN,	
	1520 -1700m asl in	1430 -1580m asl	1150 -1300m asl	
	Kisii	in Kakamega	in Kisumu	
Topography	Hills and steep	Hilly area but has	Flat land area with	
	valleys with fast	wider valleys	slower water	
	drainage		drainage	
Malaria	Epidemic prone	Mesoendemic	Hyperendemic	
Endemicity				
Prevalence of				
asymptomatic				
parasitemia	6%	30%	50%	[18]
among school				
Children				
Entomological	0.4 infective bites	16.6 infective bites	31.1 infective bites	[19]
inoculation	per person per year	per person per	per person per	
rate		year	year	
Major vectors	*Anopheles funestus*	*Anopheles gambiae*	Anopheles funestus	[20,21]
	s.l	s.s followed by	s.l and *Anopheles*	
		*Anopheles funestus*	*arabiensis*	
		s.l		
Hospitals	Marani hospital the	Iguhu and Mukumu	Kombewa hospital	
Surveyed	level four	are level four	is also the level	
(The selected	government own	government and	four government	
health facilities	hospital in the	private owned	own hospital in the	
serves both	study area with a	facilities	study area with a	
outpatient and	catchment	respectively,	catchment	
inpatient	population of about	Iguhu	population of about	
medical	19,000	areas has	23,000	
services with		population of		
sound malaria		24,000 residents		
diagnostic)				
Surveyed	Gesangora, Kiraeni	Ivonda and Iguhu	Akonya, Diemo,	
primary	and Nyasaga		Kamonye and	
schools in the			Okode	
study				

Despite the increased intervention scale up since 2006, previous epidemiological studies in these areas show Kombewa has a sustained high malaria transmission. While Marani experienced infection resurgence since 2012, Iguhu showed a positive response to interventions [13]. The major intervention against vectors in western Kenya is the use of long lasting mosquito nets, which are distributed for free through a mass distribution strategy. So far three rounds of free LLINs distribution have been completed. The first LLINs distribution was in 2006, which was followed by the second distribution in 2011, and the latest one was done in October to December 2015. The LLINs ownership has improved from as low as 24.6% and 65.8% in 2007 at Iguhu and Marani respectively to more than 80% in 2015 [13,18]. The coverage of indoor residual spray was as low as 38% between 2005 and 2010 with even lower over the recent years; none of the study sites were sprayed when this study was being done [22].

### Surveillance of asymptomatic malaria among school age children

This study describes infection transmission dynamics of the study area using school children [23]. Multiple cross-sectional surveys of asymptomatic malaria infections were done among students from nine (9) primary schools in the study areas. The study involved three schools at Marani, two school from Iguhu and four schools from Kombewa. The selected primary schools represent one-third of all within a radius of ten kilometres around the selected hospitals. These schools have been sentinel sites for more than ten years. Systematic random sampling of pupils per grade using attendance list was done in three different year of study per survey with a separate list of boys and girls. Selection of participants was done from all selected schools every month with inclusion of only those without fever and without history of related complaints. Sampling of students involved grade one to six with monthly alternation while considering equal number selected participants per grade per survey. The sampling frame was adjusted to accommodate 25 boys and 25 girls per class every month at each site. Students with fever and other malaria related symptoms were taken to nearby health facility for further management. Standard finger prick method was done to obtain thin and thick smear and Giemsa stained before microscopy examination. A minimum of 400 study participants per month was sampled from June 2015 to August 2016.

### Prospective surveillance of confirmed clinical malaria

Giemsa stained blood slides were used to diagnose existence of parasitemia in patients who visited the health facilities with suspected malaria and whom had to be sent to the laboratory for investigation. This was to confirm the positivity rate of clinical malaria in each site. During the survey, patients who were suspected to have malaria, and who agreed to sign a consent and/or assent (for minors under age of 18) form were included in the study between June 2015 to August 2016. Blood samples were collected by the standard finger-prick method and thick and thin smears were prepared on labelled slides. Blood smear tests were conducted in the hospitals as part of routine clinical practice and confirmed in the KEMRI laboratory in Kisumu. A clinical malaria case was defined as having fever on the day of the visit or 1 to 2 days earlier, parasitaemia, and one other symptoms of malaria [24]. Fever was defined as body temperature of above 37.5֯ C and hyperpyrexia was considered when the temperature was more or equal to 39֯ C. Blood slides collected from health facilities were examined at the Kenya Medical Research Institute (KEMRI) laboratory in Kisumu. Two different technicians examined the blood slides only to determine parasite presence while the third technician examined slides with disagreement. The KEMRI slides that confirmed positive cases were used to determine parasitemia rate. Additionally, all causes of outpatient visits for all age groups were counted (from the outpatient registries) along with specific numbers of diarrhoea and pneumonia cases among patients under five years of age.

### Prospective surveillance of admissions due to severe malaria

A prospective data collection of patients with features of severe malaria was done in conveniently selected hospitals. The study included all severely ill malaria patients admitted at Mukumu, Marani, and Kombewa Hospitals from October 2015 to October 2016. Their social demographics, vital signs, and observed clinical malaria complications were recorded using a checklist which captured information on routine patient care and management [25]. Severe anaemia was considered when the haemoglobin level was below 5g/dl. Days spent in hospital, microscopy results (plus parasite density) and admission outcome were also recorded.

### Review of all inpatient (IPD) malaria morbidity and mortality

Along with severe malaria surveillance, the monthly data on all causes of admission, age and the outcome (death or discharge) of admissions were also collected as from June 2015 to August 2016 for <5 children, 5–14 years and ≥15 years age groups. Data collection was done in the following hospitals: Kombewa in Kisumu county, Marani in Kisii county and Iguhu and Mukumu in Kakamega county. Iguhu and Mukumu hospitals are located in the same area close to each other (mesoendemic setting) but the former had a better OPD volume whereas the latter had a good IPD volume including all age groups.

### Review of number of outpatient (OPD) malaria cases from 2014 to 2016

Number of confirmed cases were collected from the outpatient monthly reports from January 2014 to May 2015 retrospectively and from June 2015 to August 2016. Cases were counted as they were categorized giving monthly total number of <5 children and ≥5 years of age. All causes of OPD consultations were also collected on monthly basis along with malaria cases. Moreover, during the prospective survey, a number of diarrhoea and pneumonia of OPD consultations among <5 children were also collected monthly.

### Household surveys of long lasting insecticides treated mosquito nets (LLINs) ownership and use

A total of 941 randomly selected households were surveyed for LLINs ownership and use using a pretested structured questionnaire. Pretesting of the questionnaire was done to the study site nearby villages and thereafter adjustments of the tools had to be effected. The specific number of surveyed village households were 294, 320, and 327 for Marani, Iguhu and Kombewa respectively. The sample size per village based on another similar study done in the same settings [26]. Three sub-locations were selected from each village using systematic random sampling from the local government household registry. About 100 households were sampled per each sub-location. Head of each household were interviewed on availability of the mosquito nets and use to every family member. Bed net ownership rate was measured as the ratio of the number of households with at least one bed net over the total number of households surveyed [27]. This study intended to identify the proportions of individuals who slept under LLINs. As a result, LLINs usage was defined as the percentage of individuals who reported using the mosquito net in the previous night over the total number of interviewees of the specific age category. Coverage was defined as the percentage of individuals potentially covered by LLINs, such that one LLINs for every two people for every household as per the World Health Organization guide [27]. With consent from the head of the household, observation and confirmation of LLINs availability and quality check were done.

### Statistical analysis

Proportion of malaria at the OPD was expressed at the ratio of total number of confirmed cases basing on the specified period of time (monthly or yearly) over total number of OPD consultations. Proportion of malaria admissions was computed as a ratio of all confirmed malaria admissions over the total number of admissions on the specified period of time. Clinical and asymptomatic malaria positivity rate were determined as total number people with confirmed parasitemia over total number of people tested. This led to computation of the frequency of socio-demographic characteristics and clinical features of patients admitted with severe malaria. Thus, malaria case fatality rates were computed as the total number of patients admitted and died due to malaria with or without other comorbidity over the total number of malaria admissions over the specified period of time.

The chi-square test was used to computer the differences of outpatient and inpatient proportions of malaria morbidity between the different time periods and between different age groups in different study sites. Other categorical variables were also compared by chi-square test whereas comparison of the different means were done using one way test of ANOVA. Statistical significance was considered when the p-value was less than 0.05. Bar and line graphs were used to present the number of confirmed malaria cases as well as comparison with other causes of inpatient and outpatient morbidity over a period of time per sites.

## Results

### Surveillance of asymptomatic malaria among school age children

**Fig 1:**
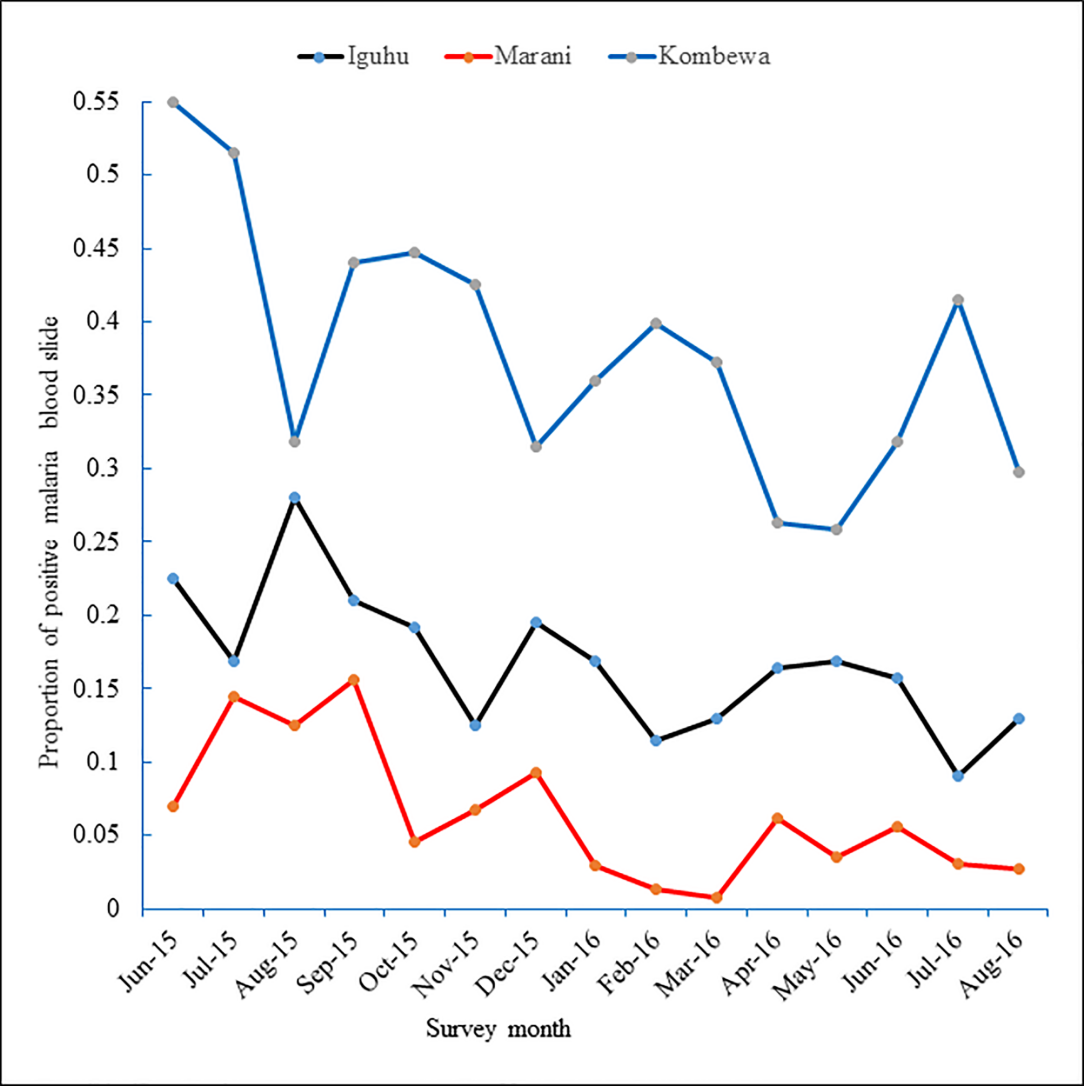
Dynamics of asymptomatic malaria parasitemia among primary school students in three study sites with different infection transmission intensity in Western Kenya from June 2015 to August 2016.

From June 2015 to August 2016, a total of 1972 and 2430 and 1759 blood slides were examined among school children living at Marani, Iguhu, and Kombewa respectively. The overall prevalence of asymptomatic malaria parasitaemia was 6.4%, 16.4% 38.3% for Marani, Iguhu, and Kombewa respectively. In June 2015, the monthly infection positivity ranged from as high as 54.9% at Kombewa while at Marani, in March 2016, the infection was as low as 0.7% (Fig 1). Of all confirmed cases, *plasmodium falciparum* was the dominant species with 95% followed by *Plasmodium malariae (4*.*5%)*, *and lastly Plasmodium ovale (0*.*5%)*.

### Prospective surveillance of confirmed clinical malaria

**Fig 2:**
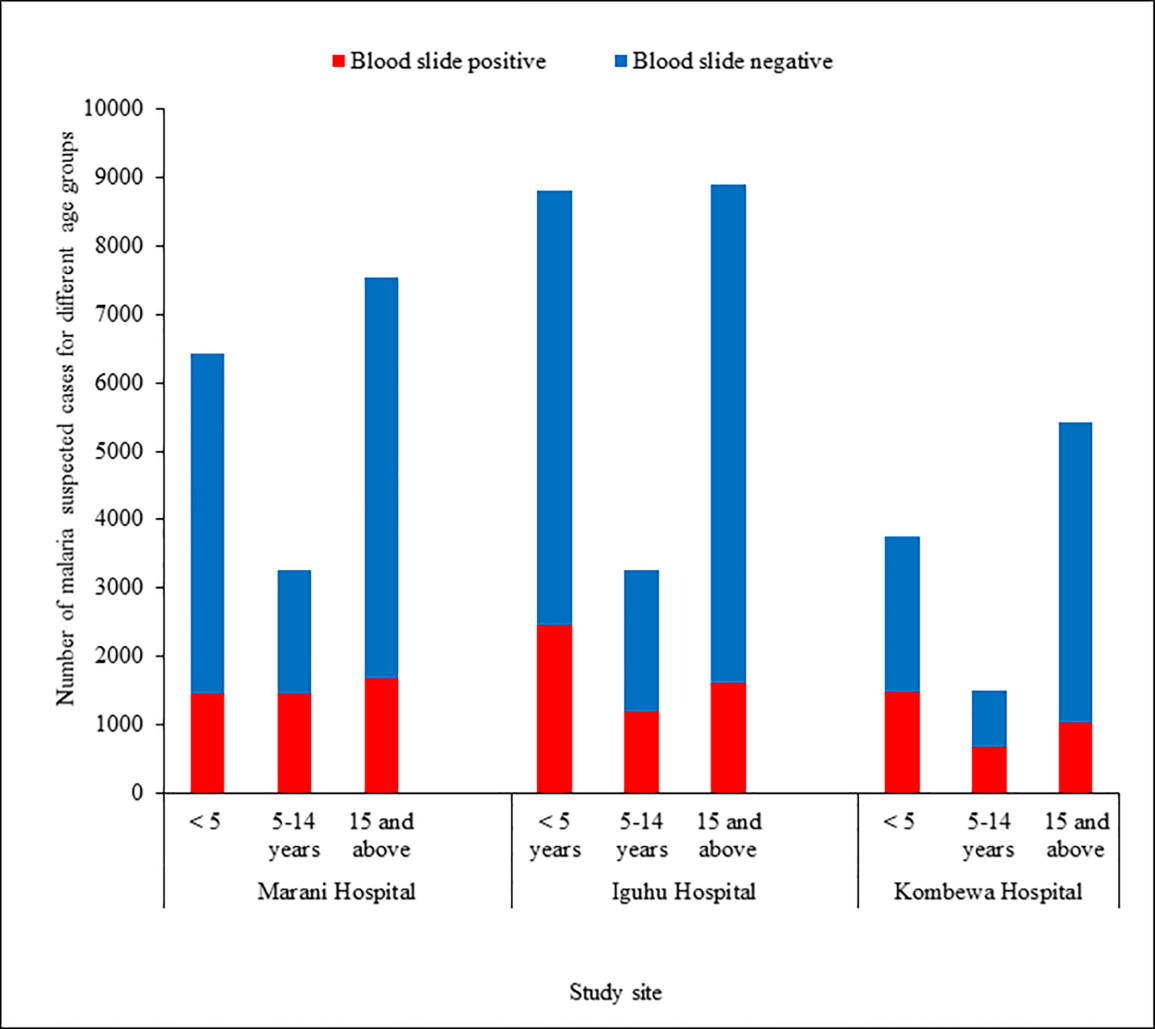
Total blood slide confirmed malaria cases among suspected patients attending outpatient departments from three hospitals located in different transmission settings of Western Kenya from June 2015 to August 2016.

**Fig 3:**
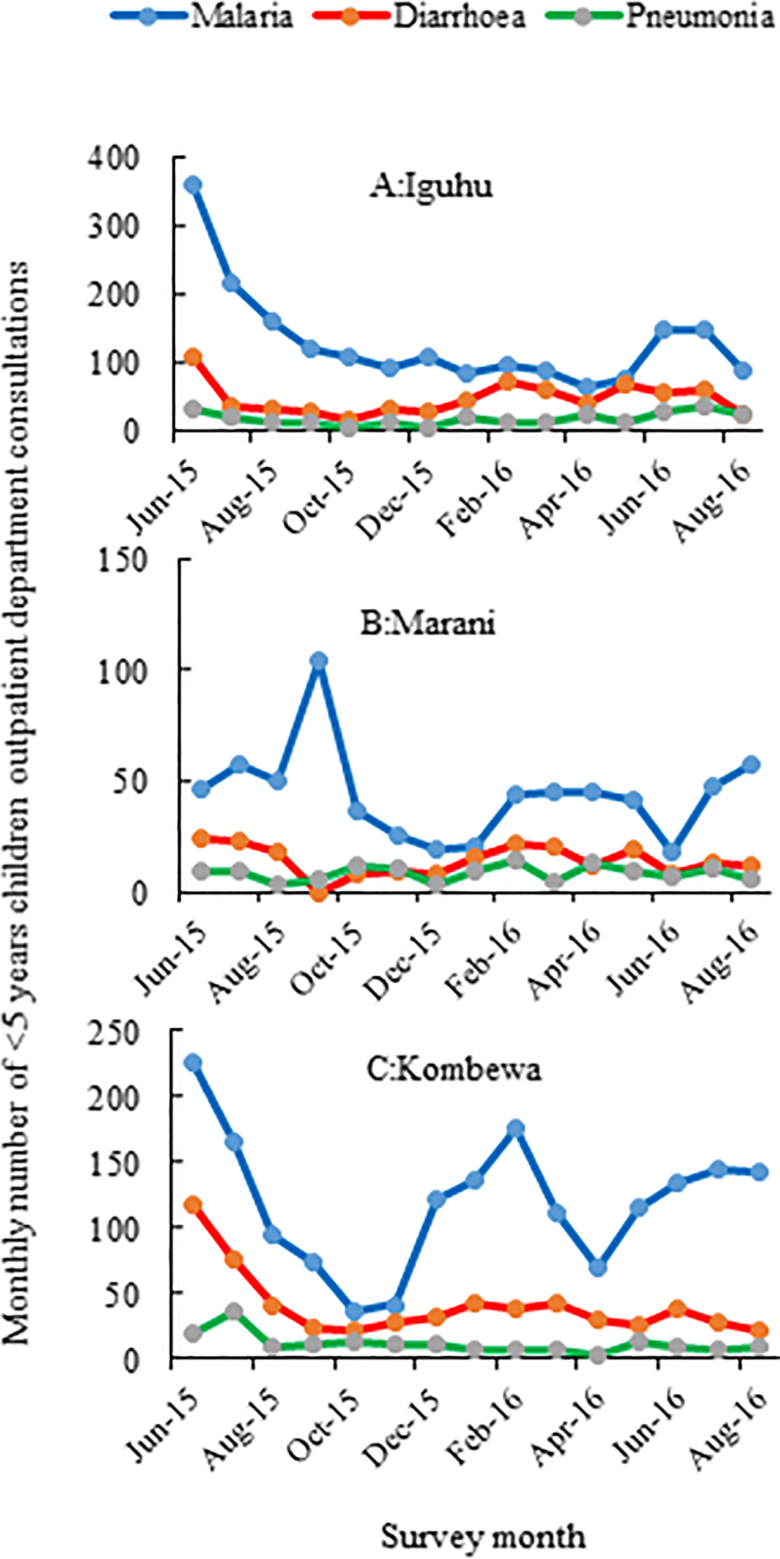
Monthly cases of confirmed malaria and other common diseases (diarrhoea and pneumonia) among under five children attending outpatient clinics from June 2015 to August 2016 in three hospital of Western Kenya.

A total of 48,892 malaria suspected patients in the 3 health facilities were screened for malaria parasitaemia. Malaria positivity rates were 26.7% (4599/17231), 24.9% (5244/20985) and 29.9% (3194/10676) at Marani, Iguhu and Kombewa hospitals respectively (χ2 = 87.9, df = 2, p<0.001). The positivity rates were highest among children of 5 to 14 years of age with 44.5% (1453/3267), 45.3% (676/1493) and 36.17% (1182/3267) at Marani, Kombewa and Iguhu hospitals respectively. The positivity rates were consistently lowest among the ≥15 years in all study sites (Fig 2). Along with the laboratory parasitological surveillance, the number of malaria cases and all causes of OPD consultations were collected within the same time frame. At Marani hospital, malaria accounted for 24.06% of all < 5 year OPD consultations whilst 18% among the ≥ 5 years (p<0.001). At Iguhu hospital however, malaria consultations were higher among the ≥ 5 years (20.77%) than the < 5 years children (17.78%) p<0.001). Whereas at Kombewa Hospital, the <5 years children accounted for 27.55% of all outpatient visits as compared to only 9.9% among the ≥5 age group (p<0.001) (Table 2). Moreover, monthly data found that malaria was consistently the leading cause of OPD consultations among the <5 children in all three hospitals followed by diarrhoea and pneumonia (Fig 3).

**Table 2:** Comparison of under-fives and over-fives proportions of confirmed malaria consultations among all outpatients (OPD) of three hospitals located in three areas with different transmission intensity of western Kenya from June 2015 to August 2016.

Hospital	Age group	OPD visits of	95% CI	p-value
	(years)	Confirmed malaria	(p_1_–p_2_)	
Marani	< 5	1457/6057 (24.06%)		
	≥5	3142/16691 (18.82%)	0.04–0.06	<0.001
Iguhu	<5	1979/11129 (17.78%)		
	≥5	2666/12835 (20.77%)	0.02–0.04	<0.001
Kombewa	<5	1785/6480 (27.55%)		
	≥5	1994/20127 (9.9%)	0.16–0.19	<0.001

p_1_ = proportion among the <5; p_2_ = proportion among the ≥5

### Prospective surveillance of admissions due to severe malaria

Clinical presentations of 1244 of severely ill malaria patients varied from site to site. For example, persistent vomiting accounted for 60.7% while prostration registered 60.6%; these were the commonest presentations at the hyperendemic site area, at Kombewa hospital. Similarly, at Marani Hospital hyperparastemia registered 40.4% and signs of prostration had 39.1%; this record emerged common at Marani epidemic prone study site. Severe anaemia malaria at the hyperendemic site was witness to only 14.3% of admissions and as low as 3% in other study areas. The number of severe malaria cases was highest among the ≥15 years old at Mukumu hospital (mesoendemic) with 51.5% and the lowest among the <5 years old with only 17.3%. Moreover, Marani hospital had approximately 70% of all severe malaria admission from the ≥5 years old with equal contribution between the 5 and 14 and ≥15 age groups. Around 63% of all severe malaria admissions at Kombewa were from <5 year old children and was lowest among children aged 5 to 14 years with only 16%. The mean length of hospital stay (days) was the longest at Kombewa (4.59±1.9) and the shortest was at Marani (2.1±2.25) (f_179.07_, df = 2, p<0.001) (Table 3).

**Table 3:** Clinical presentations of severe malaria among patients admitted in three hospitals in Western Kenya from August 2015 to October 2016.

Clinical	Variable		Hospital name		P-value
category		Marani	Mukumu	Kombewa	
		(n = 578)	(n = 254)	(n = 412)	
Age group	<5	169 (29.2%)	44 (17.3%)	259 (62.8%)	
	5–14	206 (35.6%)	79 (31.1%)	65 (15.8%)	
	≥15	203 (35.1%)	131 (51.5%)	88 (21.4%)	<0.001
Sex	Female	304 (52.6%)	130 (51.2%)	215 (52.2%)	
	Male	274 (47.4%)	124 (48.8%)	197 (47.8)	>0.05
Treatment before	Yes	254 (43.9%)	74 (29.1%)	23 (5.6%)	<0.001
admission					
Comorbidity	Yes	53 (9.2%)	09 (3.6%)	46 (11.2%)	<0.05
Admission	Mean	2.1 ± 2.25	2.7 ± 1.36	4.59 ± 1.90	<0.001
length (days)					
Clinical	Hyperpyrexia	127 (21.9%)	120 (47.2%)	118 (28.6%)	<0.001
features	Respiratory				
	Distress	26 (4.5%)	41 (16.1%)	26 (6.3%)	<0.001
	Hypoglycaemia	1 (0.1%)	2 (0.78%)	2 (0.49%)	*
	Severe anaemia	18 (3.1%)	9 (3.5%)	58 (14.3%)	<0.001
	Signs of shock	30 (5.2%)	27 (10.6%)	60 (14.1)	<0.001
	Coma	2 (0.3%)	5 (1.9%)	4 (0.9%)	*
	Prostration	226 (39.1%)	140 (55.1%)	249 (60.6%)	<0.001
				
	Convulsions	52 (8.9%)	9 (3.5%)	89 (21.6%)	<0.001
	Persistent				
	Vomiting	100 (17.3%)	65 (25.6%)	251 (60.7%)	<0.001
	Jaundice	13 (2.2%)	6 (2.3%)	3 (0.7%)	>0.05
	Hyperparastemia	234 (40.4%)	101 (39.8%)	127 (30.8%)	<0.05

*Not compared because of small number of observations

### Review of inpatient malaria morbidities

A total of 2732, 4353 and 2782 patients with different causes were admitted at Marani, Mukumu/Iguhu, and Kombewa hospitals respectively. Out of those numbers, malaria admissions accounted for 62.7%, 48.59% and 36.9% at Marani, Iguhu/Mukumu and Kombewa hospitals respectively. Generally, malaria accounted for 47% (4876/10481) of all admissions for all surveyed health facilities. Marani hospital admitted the highest proportion of patients with malaria, accounting for 63.4% of all <5 children admissions and 62.8% of all ≥5 years old inpatients (p>0.05). Malaria constituted 54.62% (721/1320), and 56.15% (657/1170) of all under-fives admissions at Iguhu/Mukumu and Kombewa hospitals respectively (p>0.05). Whereas only 38.52% (1403/3642) and 23.06% (372/1613) of all over-fives admissions were due to malaria at Iguhu/Mukumu and Kombewa hospitals respectively (p<0.001) (Table 4).

**Table 4:** Contribution of confirmed malaria cases to the inpatient admissions from three hospitals located in different malaria transmission settings of Western Kenya from June 2015 to August 2016.

Hospital	Age group	Admissions (IPD) of	95% CI	p-value
name	(years)	Confirmed malaria	(p_1_-p_2_)	(χ2)
Marani	< 5	513/809 (63.41%)		
	≥5	1210/1927 (62.79%)	-0.03–0.04	>0.05
Iguhu and Mukumu	<5	721/1320 (54.62%)		
	≥5	1403/3642 (38.52%)	0.13–0.19	<0.001
Kombewa	<5	657/1170 (56.15%)		
	≥5	372/1613 (23.06%)	0.29–0.37	<0.001

p_1_ = Proportion among <5; p_2_ = Proportion among ≥5

**Fig 4:**
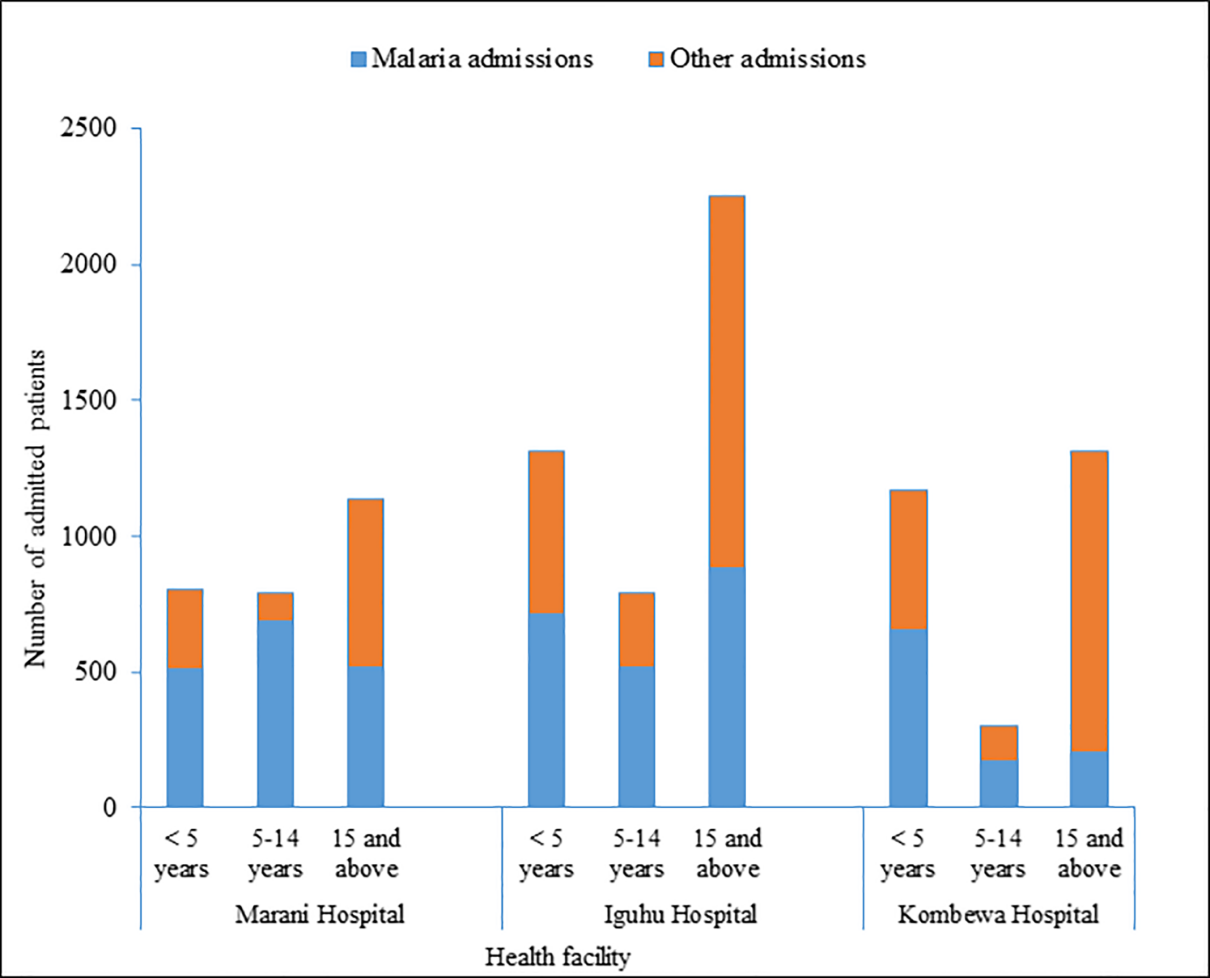
Malaria admissions and total number of admissions hospitals located in three areas with malaria transmission intensity and interventions responses in Western Kenya from June 2015 to August 2016.

Furthermore, out of all patients admitted due to malaria at Marani hospital, most of them were from the 5 to 14 age group with approximately 40% (684/1713) of all malaria admissions (χ2 = 51.45, df = 1, p<0.001). Conversely, at Iguhu/Mukumu hospital, the highest number of all malaria admissions (42% (885/2115) were from the ≥15 age group (χ2 = 11.47, df = 1, p<0.001). Kombewa hospital on contrary observed a highest number of malaria admissions (63.8% (656/1028) among the ≤5 children (p<0.001) (Fig 4).

### Review of malaria related mortalities

Lowest overall malaria related mortalities were observed at the study site with mesoendemic transmission setting (Iguhu/Mukumu) with 10 deaths per 1000 malaria admissions. However, this site observed highest death rate among the ≥15 patients (19.2 deaths per 1000 ≥15 malaria admissions) and lowest among the 5–14 with 2 death per 1000 of 5–14 years malaria admissions. Marani hospital observed 11 deaths per 1000 of all age groups of malaria admissions. Similar to Iguhu/Mukumu, Marani noted highest fatality rate among the ≥15 age group (17 deaths per 1000 ≥15 years malaria admissions); and on contrary lowest among the ≤5 children (5.8 deaths per 1000 malaria admissions of the 5–14 age group). Kombewa hospital registered the highest overall case fatality rate with 52 deaths per 1000 of all malaria admissions. This hospital, which is located in the hyperendemic setting, had the lowest fatality rate among the 5–14 age group (18 deaths per 1000 admissions of the 5–14 age group). Moreover, Kombewa showed no significant difference between the <5 children (60.9 death per 1000 admission of <5 years) and ≥5 age group (35 death per 1000 admissions (p>0.05)) fatality rates.

In comparison, malaria related death rate was higher among the <5 years old at the high transmission site of Kombewa (60.9 death per 1000 malaria <5 admissions) than the low and moderate transmission settings of Marani and Iguhu/Mukumu respectively (p<0.001). Hospitals located in the low and moderate malaria transmission settings (Marani, Iguhu and Mukumu) experienced highest malaria related death among ≥15 years old (18.6 death per 1000 admissions) than the < 15 years age group (5.7 death per 1000 admissions of the <15 years) (p< 0.001). There was no significant difference between the overall malaria related case fatality rates between Marani and Iguhu/Mukumu. Nevertheless, a higher age specific fatality rate among the 5–14 was noted at Marani when compared with Iguhu and Mukumu together (Table 5).

**Table 5:** Malaria related case fatality rates from three hospitals located in different infections transmission intensity in Western Kenya from June 2015 to August 2016.

Fatality rate	Name of the Health facility			Comparison	
(per 1000	Marani	Mukumu	Kombewa	“a” and “b”	a” and “c”
malaria	“a”	Iguhu “b”	“c”	p-value	p-value
Admissions)					
< 5 case fatality rate	(3/513)	(3/721)	(40/657)	>0.05	<0.001
	5.8	4.1	60.9		
5–14 case fatality	(7/684)	(1/518)	(3/169)	<0.05	>0.05
rate	10	2	17.6		
≥15 case fatality	(9/516)	(17/885)	(10/203)	>0.05	<0.05
rate	17	19.2	49.3		
Overall malaria	(19/1713)	(21/2124)	53/1029	>0.05	<0.001
related case	**11**	**10**	**52**		
fatality rate					

### Review of number of outpatient (OPD) malaria cases from 2014 to 2016

**Fig 5:**
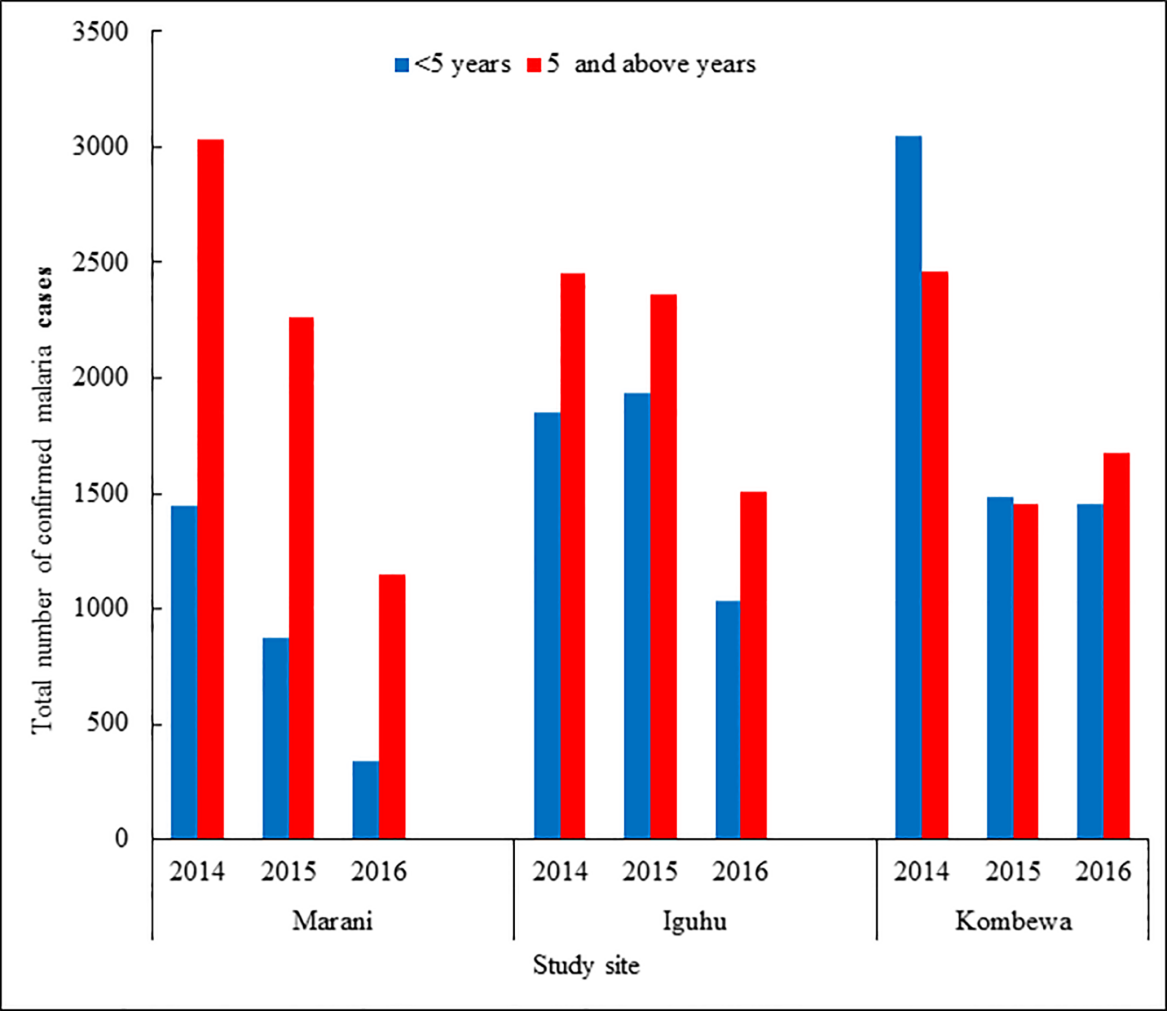
Trend of confirmed outpatient department malaria cases from 2014 through 2016 from three Hospitals in Western Kenya before and after LLINs mass distribution.

The number of Confirmed Malaria cases in three hospitals (Iguhu, Marani and Kombewa County Hospitals) was fewer by approximately 50% from 14,300 cases in 2014 to 7130 in 2016. On the contrary, Kombewa hospital showed more cases in the year just after LLINs distribution (Fig 5). There was a significant reduction of proportions of confirmed malaria out of all OPD consultation among under-fives observed between 2014 and 2016. Morbidity decline from 22.3% to 7.9% at Marani, from 23.5% to 13.2% at Iguhu, and from 32.5% to 29.5% at Kombewa (p< 0.001) was noted. Similarly, a significant reduction of OPD morbidities was observed (p<0.001) among over-fives at Marani and Kombewa. Contrarily, Iguhu experienced a significant increase of OPD malaria consultations among over-fives between 2014 (11.5% (2454/7871) and 2016 (14.8% (1510/10177) (p<0.001) (Table 6).

**Table 6:** Proportions of confirmed malaria to all outpatient department (OPD) visits from three hospitals located in areas with different transmission intensity of Western Kenya as from 2014 to 2016.

Hospital	Age group	Proportion of Confirmed malaria visits			p-value
	(years)	2014	2015	2016	Difference
			(LLINs)		2014 and 2016
Marani	< 5	** **1446/6496	872/6153	344/4313	<0.001
		(22.3%)	(14.2%)	(7.9%)	
	≥5	3033/14007	** **2162/14390	1153/11738	<0.001
		(21.7%)	(15%)	(9.8%)	
Iguhu	<5	1851/7871	1935/8461	1032/7790	<0.001
		(23.5%)	(22.9%)	(13.2%)	
	≥5	2454/21394	2364/11188	1510/10177	<0.001
		(11.5%)	(21.1%)	(14.8%)	
Kombewa	<5	3062/9422	1483/5769	1454/4923	<0.001
		(32.5%)	(25.7%)	(29.5%)	
	≥5	2464/14795	1452/12966	1564/11504	<0.001
		(16.7%)	(11.2%)	(13.6%)	

(Note: Mass distribution of LLINs to all sites was done in 2015)

### Household surveys of long lasting insecticides treated mosquito nets (LLINs) ownership and use

A total of 941 households were surveyed where 85% of them had at least one mosquito net while 43% had a net for every two individuals. The highest use of LLINs was observed among the <5 children with 90.5% (134/148), 88.9% (104/117) and 85.3% (116/136) at Marani, Iguhu and Kombewa respectively (χ2 = 0.01, df = 1, p>0.05). The lowest use of LLINs was witnessed among the 5–14 age group with 71.5% (168/235), 49.3% (105/213) and 57.6% (113/196) (Marani) at Marani, Iguhu and Kombewa respectively (χ2 = 15.03, df = 1, p<0.001). Among the ≥15 age group, the lowest use of mosquito nets was noted at Iguhu with 59.7% (191/320) whereas Marani and Kombewa had 86.7% (225/294) and 82.35 (269/327) respectively (Table 7).

**Table 7:** Household long lasting insecticide treated mosquito nets (LLINs) use and ownership survey in December 2016 in Western Kenya.

Household category	Study site			p-value
	Marani	Iguhu	Kombewa	
LLINs ownership	274/294	234/320	292/327	
	(93.19%)	(73.12%)	(89.29%)	<0.001
LLINs coverage	131/294	113/320	161/327	
	(44.56%)	(35.31%)	(49.23%)	<0.001
Any <5 child slept under	134/148	104/117	116/136	
LLINs	(90.54%)	(88.89%)	(85.29%)	0.379
Any 5–14 child slept under	168/235	105/213	113/196	
LLINs	(71.49%)	(49.3%)	(57.64%)	<0.001
Any ≥15 slept under LLINs	255/294	191/320	269/327	
	(86.73%)	(59.69%)	(82.26%)	<0.001

## Discussion

The present study investigated the current infection, morbidity and related case fatality rates of malaria in three areas with different infection transmission intensity in Western Kenya. Even though these three sites have different transmission intensity due to their different topography, altitude and climates, it is expected that with the roll out of malaria intervention through the use of LLINs, malaria infections, morbidity and mortality will drop drastically and even register no more transmission. However, the results of this study shows that the different sites have responded to the intervention differently. Whilst the intervention has reduced transmission in some sites, other sites have remained the same. This has happened despite the country’s investment in malaria strategic (2009–2018) plan of reducing morbidity and mortality by two-third between 2009 and 2018 through increase of uptake of appropriate intervention by 80% among people at risk and 100% coverage of case management among other strategic interventions [28]. Therefore enhanced monitoring of focal malaria transmission and appropriate interventions should be performed.

There was higher malaria positivity rate during surveillance of confirmed clinical cases in the hyperendemic site of Kombewa, where transmission is more perennial than in the other two sites. Transmission is mesoendemic in Iguhu/Mukumu site and epidemic prone in Marani, which has experienced epidemics in the past [29]. Higher, positivity rate among the 5–14 age group that was observed in all study sites has been as well noted in the malaria indicator surveys, in East Africa [17,30]. This could be due to increased exposure to mosquito bite as they start sleeping alone or with other children, a practice that lead to improper utilization of LLINs as observed in the current study. Monitoring clinical malaria blood slide positivity with accurate documentation and diagnosis is therefore vital to intervention evaluation.

The higher positivity rate of asymptomatic infection during the school surveys in the hyperendemic area of Kombewa did not translate into more clinical cases compared with the epidemic prone area of Marani. Malaria transmission in Marani has been historically very low [18] and therefore the people may not have enough functional immunity to malaria and with the infection of Plasmodium parasite, easily get sick. However the population in hyperendemic Kombewa and Iguhu have much exposure to sporozoites and therefore are able to tolerate carriage of Plasmodium without necessarily getting sick.

The proportion of malaria consultations at the OPD among under-fives at the epidemic prone study site was by far higher than previously reported when compared with other similar settings in Kenya [31]. This could be due to the observed infection transmission resurgence that has occurred over the recent years in this area [14]. Higher proportion of malaria consultations at the OPD among the less immune under-fives than over-fives in hyperendemic areas has been also reported in other areas [1,31–33]. On the contrary, the mesoendemic study site had higher proportion of OPD visits due to malaria among over-fives than under-fives. Studies have shown malaria burden shift from under-fives to older children and adults when the transmission intensity decreases [34,35]. The mesoendemic study site is currently also experiencing a decreasing transmission intensity [14,15].

Monthly number of OPD visits due to malaria among the under-fives was consistently higher than diarrhoea or pneumonia throughout the 15 month observations. Having higher number of OPD malaria cases increases chances of having higher cases of fatality rates than that of other childhood killer diseases. Other studies from sub-Sahara Africa also describe malaria as still an important cause of inpatient and outpatient medical consultation [31,36,37].

Severe malaria in hyperendemic settings presented mainly by life threatening persistent vomiting, convulsions and prostration. Patients presenting with severe malaria anaemia in the hyperendemic study site were less than 15% and this shows a decrease of cases when compared to the period before the intensive interventions [10,38]. Pooled data from demographic health surveys from multiple countries showed more risk of anaemia in areas with low coverage and use of mosquito bed nets [39]. The decreased severe malaria anaemia admissions in Western Kenya could be attributed to the ongoing improving bed net coverage and use [13,20,40,41].

The reported changing infection dynamics in the midst of improving LLINs coverage could potentially be catastrophic especially in the epidemic prone setting. This is consistent with other studies in the same settings, which found increasing symptomatic and asymptomatic infections in spite of expanding LLINs ownership and coverage [13,14,31].

The use of hospital records in reporting some of the results in this study may be a limitation in making some of the comparisons. Change in diagnostic and reporting accuracy, case management rate, and health seeking behaviour may also affect the comparisons. Moreover, the selected health facilities in different infection endemicity might be unrepresentative of the study area as they are few. Moreover, the current study present malaria morbidity and mortality at the level four hospitals. Therefore findings from this study may not be generalized to represent all levels of health facilities in Western Kenya.

## Conclusion

Malaria is still the major cause of admission in Western Kenya with a slight decrease of the outpatient visits. On one hand, an alarming number of the severe form of the disease among the school age children was observed in the epidemic prone setting. On the other hand, there was decreased proportion of severe malaria anaemia in hyperendemic setting of Kombewa. Mortalities were the highest among under-fives in the hyperendemic study area and among over-fifteen year olds in the mesoendemic and epidemic prone transmission settings. The case related fatality rates are comparable with the previously documented in other areas of sub Saharan Africa. Surveillance of malaria in health facilities as well as asymptomatic infections should be done and interpreted locally for better planning of interventions.
